# Response of *Caenorhabditis elegans* during *Klebsiella pneumoniae* pathogenesis

**DOI:** 10.1186/1471-2334-14-S3-P8

**Published:** 2014-05-27

**Authors:** Arumugam Kamaladevi, Krishnaswamy Balamurugan

**Affiliations:** 1 Department of Biotechnology, Alagappa University, Science Campus, Karaikudi, India

## Background

*Caenorhabditis elegans*, owing to its amenability and conserved innate immune system was recognized as an emerging model to dissect the molecular basis of many mammalian infections. *Klebsiella pneumoniae* causes severe infections in immunocompromised patients. Hence, the host response was studied using *C. elegans* during *K. pneumoniae* infection.

## Methods

Pathogenicity of *K*. *pneumoniae* and its LPS were assessed by variety of physiological and biochemical assays. The qPCR was used to analyze the regulation of innate immune genes.

## Results

*K. pneumoniae* and its LPS were lethal to *C. elegans* and required 48±5hours and 24±3hours for complete killing, respectively, with cessation of pharyngeal pumping and egg laying. Infection with *K. pneumoniae* increased the bacterial load in the intestine of host upon course of infection, which was measured as 1.5x10^3^, 2.2x10^3^, 3.6x10^4^ and 3.7x10^5^ in 4,6,12 and 24hours, respectively. This increased bacterial load subsequently disseminates oxidative stress markers in host. The level of ROS was measured to increase by 24.36597nM, 35.60517nM, 39.34052nM, and 28.24774 nM/mg of protein in 6, 12, 24, and 36 hours, respectively. Infection by *K. pneumoniae* also increased the protein carbonyls to 25.57nM, 36.14nM, 35.26nM and 38.84nM/mg of protein in 6, 12, 24, and 36 hours, respectively. *K. pneumoniae* and its LPS suppressed the expression of *pmk*-1, to l-1 and antimicrobial peptides (clec-60, clec-85, clec-87, lys-1, and lys-7) and thus succumbed the host by upregulated expression of virulent genes such as *uge*, *rmpA* and *oxyR*.

## Conclusion

The interplay between *K. pneumoniae* and *C.elegans* in the present study further provides more insights into the mechanism of host defense against this pathogen.

